# Using lithium as a neuroprotective agent in patients with cancer

**DOI:** 10.1186/1741-7015-10-131

**Published:** 2012-11-02

**Authors:** Mustafa Khasraw, David Ashley, Greg Wheeler, Michael Berk

**Affiliations:** 1 Andrew Love Cancer Centre, Geelong Hospital, Victoria, Australia; 2 School of Medicine of Deakin University, Geelong Victoria, Australia; 3 Royal Melbourne Hospital, Parkville Victoria, Australia; 4 Peter MacCallum Cancer Centre, Melbourne Victoria, Australia; 5 Mental Health Research Institute, Parkville Victoria, Australia; 6 Orygen Youth Health Research Centre and Centre for Youth Mental Health, Parkville, Victoria, Australia; 7 Department of Psychiatry, University of Melbourne, Victoria, Australia

## Abstract

Neurocognitive impairment is being increasingly recognized as an important issue in patients with cancer who develop cognitive difficulties either as part of direct or indirect involvement of the nervous system or as a consequence of either chemotherapy-related or radiotherapy-related complications. Brain radiotherapy in particular can lead to significant cognitive defects. Neurocognitive decline adversely affects quality of life, meaningful employment, and even simple daily activities. Neuroprotection may be a viable and realistic goal in preventing neurocognitive sequelae in these patients, especially in the setting of cranial irradiation. Lithium is an agent that has been in use for psychiatric disorders for decades, but recently there has been emerging evidence that it can have a neuroprotective effect.

This review discusses neurocognitive impairment in patients with cancer and the potential for investigating the use of lithium as a neuroprotectant in such patients.

## Introduction

Cognitive changes are a documented consequence of cancer therapies, including chemotherapy and radiotherapy. Indeed, the ability to inhibit cell division, the key element in cancer therapies, causes reduction in neurogenesis, which is implicated in mood and cognitive disorders. Lithium is a mood stabilizer with known neuroprotective activity, a characteristic that is thought to underpin its therapeutic efficacy. In this review, we discuss preclinical and clinical studies investigating the possibility that lithium can ameliorate the neurocognitive deterioration seen in patients undergoing cancer treatment such as cranial irradiation and chemotherapy.

The progress made in controlling systemic cancer is often hampered by relapse in the central nervous system (CNS). Systemic therapies, including both cytotoxic and biologic agents, do not achieve the same concentration in the CNS because of the blood-brain barrier. Consequently, there is a lower success rate for disease control in the CNS compared with extracranial areas. Metastatic and most primary brain tumors carry a dismal prognosis. Brain metastases are a devastating complication of cancer, and have been designated as an area of unmet need by the US Food and Drug Administration.

Radiotherapy is delivered to the brain for the palliative treatment of primary brain tumors and brain metastases, and also for treatment to prophylactically decrease the occurrence of CNS relapse in selected patients in diseases such as small cell lung cancer (SCLC) and certain hematological malignancies with known high rates of CNS relapse. Brain radiotherapy can lead to cognitive impairment and mood symptoms, which can further decrease quality of life in patients with limited expected survival. Chemotherapy drugs, such as doxorubicin, are similarly associated with cognitive side effects [1,2]. Patients who achieve long-term remission may struggle to return to normal life and functioning because of cognitive impairment.

### Neurocognitive impairment in patients with cancer

In broad terms, brain tumor treatment is multimodal, with surgery, radiotherapy, and chemotherapy being involved. All three treatments may affect the neurocognitive outcomes [3]. In addition to its use in patients with cancer involving the CNS, brain radiotherapy is also used as prophylaxis in patients with limited stage SCLC who achieve good extracranial disease control.

### Chemotherapy and neurocognition

'Chemo brain' has been studied in women with breast cancer, and also in other malignancies such as colon and non-small cell lung cancer (NSCLC). These studies seem to show a decline (sometimes transient) in cognitive function, after chemotherapy or haemopoietic stem-cell transplantation, particularly executive functions and short-term memory [4,5]. Using functional neuroimaging, abnormal activity of the frontal cortex, cerebellum, and basal ganglia has been shown in breast-cancer survivors as long as 5 to 10 years after chemotherapy [6]. Some studies with follow-up periods of more than 20 years show that women with breast cancer perform worse, on average, than random population controls on neuropsychological tests [7]. However, some patients may perform less well than controls even before chemotherapy. It is not known whether a component of the pre-treatment cognitive changes is paraneoplastic. Neuropsychological training may improve cognitive performance,[8] and pharmacologic intervention with agents such as modafinil may also be helpful [9].

Acute cognitive change induced by specific chemotherapy drugs is rare, with the exception of ifosfamide, which causes encephalopathy in approximately 12% of patients [10]. The delirium usually resolves within a few days. High-dose methotrexate may cause delirium with encephalopathy several days after the infusion, which lasts for a few days [11]. Indeed, methotrexate, whether given intravenously or intrathecally, is one of the few chemotherapeutic agents that cause significant cognitive dysfunction.

### Radiation and neurocognition

Radiation mediates neurocognitive effects by affecting glial cells, neural stem and progenitor cells [12,13], and the vascular structures [14,15]. Cranial irradiation delivered to mice has been shown to reduce neural proliferation, translating to long-term reduction in neurogenesis [16,17]. Reduction in neurogenesis is documented as a core component of the pathophysiology of depression and dementia, and many treatments for depression may promote neurogenesis[18]. Stress that can precipitate mood disorders can additionally produce neuronal atrophy and reduction of neurogenesis [19].

Radiotherapy is a significant factor in the neurocognitive decline of patients with cancer with brain metastases. Chronic changes in cognitive function not preceded by delirium can result from brain radiation, sometimes enhanced by chemotherapy, particularly with methotrexate [3].This is particularly evident in long-term survivors of radiosensitive malignancies, such as CNS lymphomas and some cases of SCLC.

Brain radiotherapy is commonly used as a palliative treatment for patients with brain metastases, but also in diseases with a relatively good prognosis, such as medulloblastoma, ependymoma, germinoma, and acute lymphoblastic leukemia. As mentioned above, prophylactic cranial irradiation is also used in certain clinical situations, such as with limited-disease SCLC and good extracranial response to therapy.

Acute encephalopathy may result from radiation with fractions of 300 cGy or more to a large CNS treatment field. This can result in, or contribute to, other causes of delirium. Patients who have large tumors with increased intracranial pressure, and who are irradiated with high doses per fraction, may develop acute encephalopathy and even brain herniation after the initial few treatments [20]. Although there are no data from studies on this, corticosteroids are used in practice either to prevent or ameliorate symptoms.

Somnolence syndrome is a common intermediate side effect of cranial radiotherapy, which occurs approximately 6 weeks after treatment, and manifests as lethargy, increased sleepiness, poor attention or subtle memory changes, and altered temperament [21]. Somnolence syndrome occurs in approximately 60% of children treated with cranial irradiation for acute lymphatic leukemia, and in up to 80% of adults who are treated with cranial irradiation for brain tumors [22]. Somnolence syndrome usually occurs approximately 6 weeks after treatment, and manifests as lethargy, increased sleepiness, and poor attention or memory. There have been some reports of an associated fever [23]. In adults, the condition tends to be subtler, with memory changes and altered temperament. It is self-limiting, and does not seem to be linked to later sequelae. It is thought to be due to acute demyelination affecting neural signal transmission. Large doses of dexamethasone given during radiotherapy has been suggested to ameliorate this, but this is not routine practice [23].

Neurocognitive complications of cranial radiation are among the most distressing symptoms for patients, families, and care providers. In children who have craniospinal irradiation for medulloblastoma or germinoma, or more focal radiation for tumors such as ependymomas, gliomas, or craniopharyngiomas, the late effects are marked. Commonly, there is a decline in intelligence quotient (IQ) (full-scale, verbal and nonverbal domains), which tends to be more marked in patients who have a younger age at treatment; larger volumes of brain irradiated, particularly the temporal lobes; higher premorbid IQ; hydrocephalus requiring a shunt; and intrathecal chemotherapy in addition to radiotherapy. Children often have an initial improvement after the completion of therapy, but a later decline occurs 12 to 24 months later [4]. The decline clearly affects school completion and vocational achievements in later life. In older children, impaired short-term memory, processing speed, and shortened concentration span are common, and these problems progress with age.

The desire to reduce neurocognitive complications has driven many recent studies. Historically, children treated before the age of 2 years were often institutionalized because of their iatrogenic impairments. In both adults and children, dose, brain volume exposed, and fraction size are strong determinants of the outcomes. It is now well known that children receiving brain irradiation experience less severe neuropsychologic toxicity when treated with 23.4 Gy, rather than 36 Gy cranial irradiation, and that older children are less likely to develop cognitive impairment than children who are younger at the time of irradiation, suggesting greater vulnerability of the developing brain [24].

The use of cranial irradiation is linked to problems with long-term memory and concentration [15]. Modern radiotherapy techniques allow for sparing of sensitive areas of the CNS. Techniques such as intensity-modulated radiation therapy (IMRT) allow sparing of crucial structures such the optic chiasm or the pituitary gland. Sparing of the hippocampus, which has a crucial role in short-term memory, can also be achieved with the use of IMRT. This approach can still deliver acceptable target coverage and homogeneous delivery to the tumor area in the case of brain metastases [25]. Obviously, short-term memory is only one of the cognitive aspects affected by hippocampal damage. Exposure of other parts of the brain, such as the frontal and prefrontal cortex, may have a more subtle effect. Declines in attention, executive function, and motor, language, and general intellectual skills have been reported. Therefore, prospective well-controlled studies are needed to validate the role of hippocampal sparing. This question is currently under investigation in a randomized controlled phase II cooperative group trial to test the hypothesized neurocognitive benefit.

Additionally, there is a suggestion of a more rapid progression of dementing illness in patients receiving cranial irradiation, although the number of long-term survivors is limited [15]. There is also a risk of increased frequency of cerebrovascular accidents and small-vessel disease, thought to be due to intimal thickening of the cerebral arteries. Other late changes that can occur are the development of small cavernomas within the white matter, which may occasionally cause a low-pressure bleed, of little clinical significance.

Depression is another significant problem in this setting, and may be linked to reduction in neurogenesis. Indeed, it is possible to block the efficacy of antidepressants in some, but not all, models of antidepressant efficacy by impeding neurogenesis using cranial radiation [26]. This suggests that there are neurogenesis-dependent and neurogenesis-independent mechanisms of antidepressant action.

### Lithium and neurocognitive dysfunction

Lithium is a standard part of the management of moderate to severe bipolar disorder and schizoaffective disorders, with a well-understood toxicity profile [27-29].

#### *Preclinical data on the neuroprotective effect of lithium *(3)

Lithium exerts neuroprotective effects and is associated with less cognitive loss in various brain-injury models, including after cranial irradiation [30,31]. In addition, neural stem/progenitor cells positively respond to lithium treatment under basal conditions [32,33]. In addition to evidence from animal studies, neuroimaging research in humans supports the observation that lithium exerts neuroprotective effects. One study used three-dimensional magnetic resonance imaging and brain segmentation to evaluate increases in the effect of lithium on grey-matter volume in patients with bipolar mood disorder. The use of 4 weeks of lithium treatment was shown to increase brain grey-matter content [34] and hippocampal volume [35]. The authors concluded that increases in grey matter probably occurred as a result of neurotrophic effects.

Lithium was found to protect irradiated hippocampal neurons in mice from apoptosis, resulting in better performance in learning and memory function [31]. Lithium is known to reduce oxidative stress, specifically via the glutathione system [36]. In bipolar disorder, lithium has been shown to prevent the loss of cortical grey matter that occurs as part of the neuroprogressive cascade in the disorder [37].

Whole-brain irradiation delivered to mice has been shown to reduce neural proliferation in the dentate gyrus subgranular zone, translating to long-term reduction in neurogenesis [38]. The number of neurons in the brain is controlled by production of new neurons and neuronal death. Glutamate, glucocorticoids, and haloperidol reduce neural progenitor proliferation in cerebellar granule cells [39], whereas lithium prevents the loss of proliferation induced by these agents. Microangiopathic and capillary loss in the treated areas, compounded by intimal thickening in the larger vessels, leads to reduction in vascularity and in white-matter damage [40,41].

Neural progenitor proliferation in the developing and adult brain plays a prominent role in the production of new neurons. One of the main targets of radiation to treat the observed neurocognitive effects seems to be the glial cells [42]. Oligodendrocyte loss in particular seems to affect the development of myelination of the damaged nerves [43]. Other major targets are neural stem and progenitor cells and the vascular structures [42]. Lithium increases the recovery of proliferating neural stem and progenitor cells in the dentate gyrus, and reduces the incidence of radiation-induced gliosis. In addition, neural stem and progenitor cells respond positively to lithium treatment under basal conditions [32] and in brain-injury models [33].

Lithium-induced neural progenitor proliferation *in vitro *suggests that similar effects might occur *in vivo*, and this action could also be related to its clinical efficacy [39]. In animal studies, the effect of lithium treatment is partly mediated by inhibiting inflammation and by promoting proliferation and survival of neural stem and progenitor cells [44]. Lithium was shown to increase progenitor, rather than stem-cell, proliferation in both non-ischemic and ischemic rat brains [44].

The nt/β-Catenin pathway regulates cell-fate decisions during development in vertebrates and invertebrates. Glycogen synthase kinase (GSK)-3 has been shown to be an essential mediator of neural progenitors during brain development. The Wnt ligand is a secreted glycoprotein that binds to receptors, leading to displacement of the multifunctional kinase GSK-3β from the the adenomatous poliposis coli protein (APC)/axin/GSK-3β complex. In the absence of Wnt-signal (off-state), β-catenin, an integral cell-cell adhesion adaptor protein and transcriptional co-regulator, is targeted for degradation by the APC/axin/GSK-3β-complex [45]. Lithium is an activator of β-catenin signaling, and may overcome inhibition of β-catenin signaling [46]. Lithium may also exert some of its neuroprotective actions via inhibition of GSK-3β [47]. Bcl-2 and Bax are apoptosis-related genes. Lithium induces a decrease in Bax protein levels and causes an increase in the Bcl-2/Bax ratio [48]. Long-term lithium treatment suppresses p53 and Bax expression, but increases Bcl-2 expression [48]. Activation of WNT signaling by lithium chloride, which inhibits the negative regulator of the WNT/β-catenin pathway (GSK3β), reduces astrocytic activation in neurodegenerative diseases, and is thought to do so through its ability to induce WNT signaling [46]. The neurotransmitter dopamine can exert its effects by acting on a lithium-sensitive signaling cascade involving Akt/PKB and GSK-3 [49]. In mouse striatum, lack of the dopamine transporter results in inactivation of Akt and concomitant activation of GSK-3α and GSK-3β, but these changes are effectively reversed by lithium [49]. Lithium additionally seems to block the excitotoxicity that is mediated by increased levels of intracellular calcium [50]. This is a pathway to apoptosis, [51-53] mediated by glutamate, and is a replicated finding in mood and cognitive disorders [54].

The Morris water maze (MWM) is a test of spatial learning for rodents, in which the rodents rely on distal cues to navigate from start locations around the perimeter of an open swimming arena to locate a submerged escape platform [55]. Lithium was found to protect irradiated hippocampal neurons in mice from apoptosis, resulting in better performance of rats in the MWM, reflecting better learning and memory function.

### Clinical data on the potential neuroprotective effect of lithium

There are limited prospective clinical data on the use of lithium as a neuroprotectant. Several small imaging studies have shown that patients with bipolar disorder treated with long-term lithium therapy have fewer structural changes on brain imaging compared with patients with bipolar disorder of at least 2 years in duration who received lithium for less than 3 months. Patients with bipolar disorder who were not treated with lithium were found to have smaller left hippocampal volumes than controls (corrected *P*<0.05). The study included 17 patients with bipolar disorder who had at least 2 years of lithium therapy, compared with 12 patients with bipolar disorder who had less than 3 months of lifetime lithium exposure. The group treated with lithium had hippocampal volumes similar to those of 11 healthy controls and of young, lithium-naïve patients [56]. In a similar study, measurement of left prefrontal N-acetyl aspartate (NAA) levels was performed using magnetic resonance spectroscopy at 1.5 T. The study included 27 participants treated with lithium, 16 participants not treated with lithium (<3 months exposure) and 21 healthy controls. The non-lithium group had lower prefrontal NAA levels than the lithium-treated group (*P*<0.01) or control group (*P*<0.05) [57].

In a large observational cohort study in Denmark, Kessing and colleagues showed that patients taking lithium were more likely than the general population to develop dementia; however, for those who continued to take lithium, the rates fell to those of the general population [58]. It is unclear whether this reflects drug effects, or is a proxy of disease-related effects, as people with mood disorders are at increased risk of developing dementia compared with the general population. This effect was not seen in the population taking anticonvulsants. A follow-up study published by the same group in 2010 showed that continued treatment with lithium was associated with a reduced rate of dementia in patients with bipolar disorder, in contrast to continued treatment with other drugs such as anticonvulsants, antidepressants, or antipsychotics [59]. A meta-analysis of lithium on cognitive performance found minor negative effects on cognition [60].

Long-term lithium treatment was also investigated in amnestic mild cognitive impairment as part of a study that randomized 45 participants to receive lithium (0.25 to 0.5 mmol/l) (n = 24) or placebo (n = 21) in a double-blind trial [61]. Lithium treatment was associated with a significant decrease in CSF concentrations of P-tau (P = 0.03). Tau proteins are proteins that stabilize microtubules. When tau proteins are defective, and no longer stabilize microtubules properly, they can result in dementias. The study reported better performance on the cognitive subscale of the Alzheimer's Disease Assessment Scale and in attention tasks [61].

In an open-label study, the effects of administering lithium carbonate to patients with Alzheimer's disease for up to 1 year were evaluated [62]. No cognitive benefits were found in the patients who completed the study. In a different placebo-controlled, single-blind study, lithium was used to treat patients with mild Alzheimer's disease for 10 weeks. Lithium treatment was not found to have significant benefits on either cognitive performance or CSF concentrations of disease-related biomarkers [63].

There has been one early-phase study in which lithium was used as a neuroprotectant, which was presented in abstract form at the 2007 American Society for Therapeutic Radiology and Oncology [64], and updated at the 2008 annual meeting of the Society of Neuro-Oncology [65].

## Conclusions

Neuroprotection may be a viable and realistic goal in preventing neurocognitive sequelae in patients with cancer, especially in the setting of cranial irradiation. There are two types of pathological processes amenable to intervention that could be targeted: 1) normal physiological processes that happen in excess, such as excitotoxicity, pruning. or excessive apoptotic activity [48] and 2) failure of trophic processes, such as reduced neurogenesis, senescence of progenitor cell generation, and differentiation. Such an approach would involve regulating the processes of growth and regeneration, and the rescue of brain cells that may be at risk of damage or even death [66].

Lithium is an agent that has been in use for psychiatric disorders for decades, providing us with a wealth of experience with this 'old' medication that has the potential for a novel indication. Nevertheless, there remain many unanswered questions. Because lithium may reduce free radical damage, which is one of the primary mechanisms of action for radiotherapy, it may reduce the efficacy of radiotherapy. There is also a theoretical concern that if the neural proliferating cells are stimulated during radiation treatment, this may actually increase the neural damage by reducing the repopulation pool.

In many studies of the biochemical and behavioral models, not only has lithium been found to be neuroprotective, but also antidepressants, atypical antipsychotics, and many substances such as vitamin A. However, none has shown a significant effect size in subsequent adequately powered human clinical trials for this indication. To date, there is no evidence that a pharmacological intervention could prevent or reduce radiation-induced somnolence syndrome or late neurocognitive impairment. In palliative patients, whose median survival may be measured in weeks, a significant proportion of survival time is affected by somnolence syndrome. Neurocognitive decline adversely affects quality of life, meaningful employment. and even simple daily activities [67,68]. Lithium has shown promise in preliminary studies, but if its benefit as a neuroprotectant in patients receiving cancer treatment is confirmed, it may ultimately lead to improvement in the quality of life for these patients.

## List of Abbreviations

CNS: Central nervous system; CSF: Cerebrospinal fluid; GSK: Glycogen synthase kinase 3; IQ: Intelligence quotient; IMRT: Intensity-modulated radiation treatment; MWM: Morris Water Maze; NAA: N-acetyl aspartate; SCLC: Small-cell lung cancer

## Competing Interests

The authors have received grants from the Victoria Cancer Agency and The Viertel Charitable Foundation to undertake research investigating the neuroprotective effect of lithium in patients receiving cranial irradiation.

## Authors' contributions

MK and MB both conceived and designed the study, and wrote the manuscript. All the authors took part in the literature review. All the authors have read and approved the final manuscript.

## Pre-publication history

The pre-publication history for this paper can be accessed here:

http://www.biomedcentral.com/1741-7015/10/131/prepub
